# Compared With a Nasointestinal Route, Pre-operative Enteral Nutrition *via* a Nasogastric Tube Reduced the Incidence of Acalculous Acute Cholecystitis After Definitive Surgery for Small Intestinal Fistula

**DOI:** 10.3389/fmed.2021.721402

**Published:** 2021-08-17

**Authors:** Zheng Yao, Weiliang Tian, Xin Xu, Risheng Zhao, Yunzhao Zhao

**Affiliations:** ^1^Department of General Surgery, Jiangning Hospital, Nanjing, China; ^2^Department of General Surgery, Jinling Hospital, Nanjing, China

**Keywords:** acalculous acute cholecystitis, intestinal fistula, surgery, nasogastric tube, nasointestinal tube

## Abstract

**Purpose:** This study aimed to investigate the difference in the efficacy of pre-operative enteral nutrition (EN) *via* a nasogastric tube (NGT) and pre-operative EN *via* a nasointestinal tube (NIT) in reducing the incidence of post-operative acalculous acute cholecystitis (AAC) after definitive surgery (DS) for small intestinal fistulas.

**Methods:** Patients with a small intestinal fistula, who had a DS for the disease between January 2015 and March 2021, were enrolled in this study. They were divided into the NIT group and the NGT group based on the pre-operative routes of feeding they received. The clinical characteristics of the two groups were analyzed, and the incidences of post-operative AAC in the two groups were evaluated.

**Results:** A total of 200 patients were enrolled in the study, 85 in the NGT group and 115 in the NIT group. Thirty-one patients developed post-operative AAC (8 in the NGT group and 23 in the NIT group). The incidence of post-operative AAC was 15.5%. EN *via* the NGT route was associated with a reduction in the incidence of post-operative AAC (adjusted HR = 0.359; 95% CI: 0.139–0.931; *P* = 0.035).

**Conclusion:** Pre-operative EN *via* the NGT may reduce the incidence of post-operative AAC in patients who received a DS for small intestinal fistulas.

## Introduction

Acute acalculous cholecystitis (AAC) is defined as an acute inflammation of the gallbladder without gallstones. Comparing to the incidence of AAC in non-critically ill patients, the incidence of AAC is higher in critically ill patients (1, 2). AAC is associated with high patient morbidity and mortality. The mortality rate is up to 50% (3–5). The potential risk factors of AAC are major surgery or trauma (6), burns (7), sepsis (8), and the administration of total parenteral nutrition (9). Bile stasis and gallbladder wall ischemia are the two possible pathophysiological processes leading to AAC (10).

Fasting, fluid deprivation, and enteral nutrition (EN) are essential treatments before a definitive surgery (DS) for intestinal fistulas. A nasointestinal tube (NIT) and a nasogastric tube (NGT) are the two main routes for administering EN in clinical practices. For patients receiving the EN *via* an NGT, the formula reaches the stomach and the duodenum before entering the lower gastrointestinal tract. This process of the entry of food is closer to the natural physiology of digestion while comparing to the NIT. Besides, this method does not interfere with the release of hormones at the duodenum and proximal jejunum, for instance, the release of cholecystokinin (CCK) (11). In contrast, the EN *via* the NIT works by continuously pumping the enteral feeding formula to the intestine directly at a constant speed. This method causes little stimulation to the upper gastrointestinal tract, which helps to reduce the fistula output. It also reduces the production and secretion of gastrointestinal hormones, including CCK. The reduction in the CCK level may lead to bile stasis, which greatly increases the risk of AAC (12). In addition, a DS is relatively difficult to perform for some small intestinal fistulas, and intraoperative hemorrhage might occur during the operation. Massive intraoperative blood loss will lead to severe hypoperfusion and severe surgical strike. Thus, a DS may contribute to ischemia, the other major risk factor for AAC. In theory, small intestinal fistulas patients receiving pre-operative EN *via* the NIT may have a higher risk of post-operative AAC than the patients receiving pre-operative EN *via* the NGT.

This study retrospectively investigated the post-operative characteristics of patients receiving a DS for the small intestinal fistula. It also evaluated the efficacy of the pre-operative EN *via* the NGT in reducing the risk of post-operative AAC.

## Materials and Methods

This was a retrospective cohort study performed at two tertiary hospitals in Nanjing, China.

### Study Design

From January 2015 to March 2021, patients who received a DS for a small intestinal fistula were enrolled in the study. The exclusion criteria were as follows: (1) patients younger than 18 years old; (2) patients having potential infections besides the small intestinal fistulas, such as duodenal fistulas, colonic fistulas, and previous severe pancreatitis; (3) patients whose gallbladder was removed before or during the DS; (4) patients received percutaneous cholecystostomy (PC) pre-operatively, and the cholecystostomy tube was not removed before the surgery; and (5) cases with missing clinical data. The patients were divided into the NIT group and the NGT group based on their pre-operative feeding routes. The clinical data of the two groups were collected and evaluated.

Patients were followed up until discharge. The primary outcome was the incidence of post-operative AAC. The secondary outcomes were the characteristics of AAC, including signs, symptoms, and post-operative length of stay (LOS).

### Criteria for Using NIT or NGT

Previously, no standard criteria were used for deciding the pre-operative route of feeding. Every patient revived an NIT. The rationale behind this decision was that the NIT feeding causes less stimulation to the GI tract than the NGT feeding does, and therefore, helps to control the fistula output.

Later, the output was no longer a concern as the application of chyme reinfusion became prevalent. As a result, the usage of the NGT gradually increased, especially in patients with a shorter total length of the intestine because this method maximizes the use of length of the digestive tract.

### Perioperative Treatment and the DS

The DS for fistulas was planned when patients met the following conditions: (1) Basic metabolic panel, complete blood count, and infection indicators (CRP and PCT) were normal for at least 1 month. (2) The patients had a BMI > 18.5 kg/m^2^ and had normal physical strength. (3) The patients had no fever, abdominal distention, or vomiting for at least 1 month. (4) It had been at least 2 months interval between the last abdominal operation and the planned DS (or 4 months in the case of patients with an enteroatmospheric fistula). (5) Ideally, the total EN was given for at least 2 months; or EN plus parenteral nutrition (PN), and the EN should provide more than 1,000 kcal/day without the use of somatostatin.

Two surgical teams, both led by Zhao Yunzhao, MD, Ph.D., performed the DSs in the two hospitals. A combination of sharp and blunt dissection methods was used to open the digestive tract gradually in the surgery. During this process, the grade of abdominal adhesion and the length of the small intestine were evaluated. The side-to-side anastomosis was performed using a curved intraluminal stapler (Ethicon, Endo-Surgery, NM, USA). The patient with an open abdomen or enteroatmospheric fistula would receive a one-stage abdominal closure with the component separation technique and onlay mesh repair using a Cook Biodesign advanced tissue repair device (Cook Medical Inc., IN, USA).

The post-operative fluid management was based on the changes in post-operative vital signs, the mean arterial pressure, the central venous pressure, and the lactic acid level. We aimed to raise the patient's hemoglobin to >100 g/L and the albumin to >30 g/L within the first 48 h post-operatively (with proper diuresis allowed).

Post-operative EN was applied when the drainage of the gastrointestinal decompression was <100 mL/day, and the patient already had bowel movements. The initial rate of EN was set at 20 mL/h (using a formula of 1.0 kcal/mL) and gradually increased to total EN. During the course of post-operative treatment, the PN would be tapered off when the patient could tolerate an EN intake of >1,000 kcal/day.

### Diagnosis and Treatment of AAC

Patients who had an unexpected fever, abdominal pain, abdominal distension, and increased infection indicators were suspected of having AAC. A CT scan would be performed then. If the imaging examination confirmed a full gallbladder and the thickness of the gallbladder wall was >3 mm, AAC would be diagnosed. After AAC was diagnosed, meropenem (1 g every 8 h by intravenous infusion) and metronidazole (0.5 g every 12 h by intravenous infusion) would be initiated. Besides the antibiotics, an ultrasound or CT-guided PC would be used to treat AAC.

### Data Collection and Analyses

Data on demographics, routine blood tests, and basic metabolic panel results of the patients were recorded within 3 days before the surgery. The intraoperative bleeding and the duration of the DS were obtained from the operation records. Post-operative albumin infusion, blood transfusion, and arterial blood gas analysis results were extracted from the post-operative ICU records. In the patient diagnosed with AAC, the bile drained from the PC was cultured for bacteria growth. The signs and symptoms of AAC were identified from the patient's clinical records.

Organ injury was defined as a score of >2 using the Sequential Organ Failure Assessment scoring (13). According to this scoring system, acute hepatic injury is defined as a total bilirubin level of >33 μmol/L (13). Acute kidney injury is defined as a creatinine level of >176 μmol/L (13). The abdominal adhesion was evaluated according to the assessment method established by Hobson et al. (14). The abdominal adhesions were classified into the following degrees: Degree I = no adhesion; Degree II = minimal adhesions localized to one or two areas; Degree III = diffused adhesions, but not extensive; Degree IV = diffused, extensive adhesions, easily lysed; Degree V = diffused, extensive, dense adhesions, difficult to lyse.

All statistical analyses were performed using the SPSS 26.0 (IBM Analytics, NY, USA). A Mann–Whitney *U*-test was used to compare continuous variables. The Fisher exact-test was used to compare categorical variables. K–M and multivariate regression analyses were used to compare the efficacy of different methods in reducing AAC. A *P*-value < 0.05 indicated a statistically significant difference.

## Results

### Population

**Figure 1:**
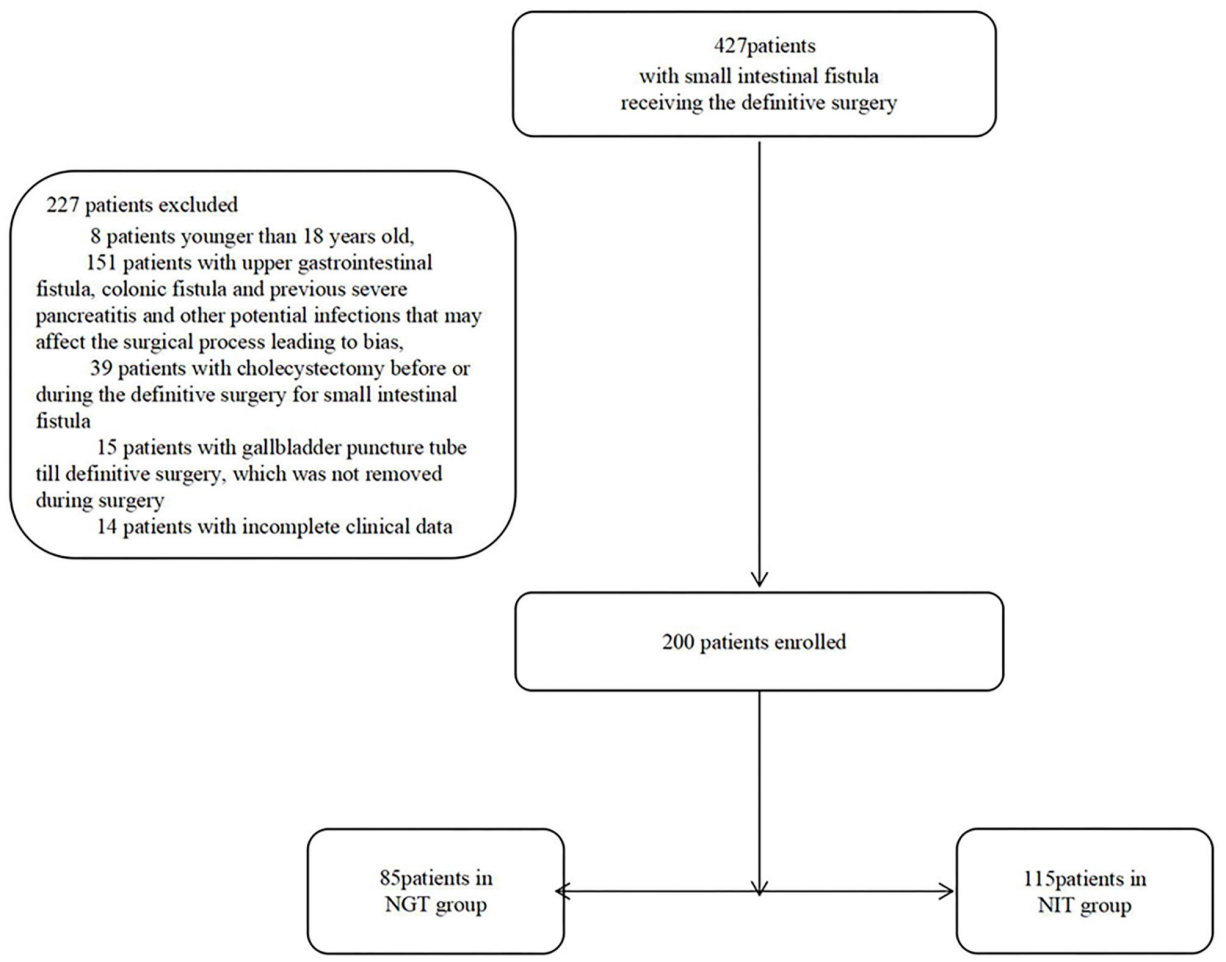
Patients and grouping.

From January 2015 to March 2021, 200 patients were enrolled (Figure 1). Of the 200 patients, 85 were in the NGT group, and 115 were in the NIT group (Table 1). Of the 200 patients, 98 (49%) were women. The NGT group had a shorter distance from the Treitz ligament to the fistula, and a shorter total length of the small intestine than the NIT group did (Table 1). However, no significant differences were found for other characteristics between the two groups.

**Table 1:** Baseline characteristics.

	**NGT group (*n* = 85)**	**NIT group (*n* = 115)**	***p***
Female, No. (%)	44(51.76)	54 (46.95)	0.501
Age, No. (%)			0.369
<60	78 (91.76)	101 (87.83)	
More than 60	7 (8.23)	14 (12.17)	
BMI, kg/m^2^, (mean ± SD)	19.73 ± 1.24	19.44 ± 1.23	0.109
Etiology, No. (%)			0.711
Trauma	29 (34.11)	32 (27.83)	
Obstruction due to previous surgery^*^	26 (30.58)	40 (34.78)	
Mesenteric thrombosis	12 (14.11)	14 (12.17)	
Spontaneous perforation due to crohn's disease	18 (21.18)	29 (25.22)	
Length from treitz to location of (the first) fistula, cm, (mean ± SD)	178.24 ± 42.37	209.22 ± 78.99	0.001
Number of fistula, No. (%)	1 (1–1)	1 (1–1)	0.485
High output, No. (%)	60 (70.58%)	72 (67.65%)	0.874
Pre-operative parenteral nutrition required, No. (%)	7 (8.24)	5 (4.35)	0.367
Interval from fistula occurred to admission, days, (median, IQR)	25 (21.75–30)	25 (22–32)	0.834
Interval from fistula occurred to definitive surgery, days, (median, IQR)	140 (124–148.5)	137 (129–146)	0.887
Pre-operative albumin, g/L, (median, IQR)	36.4 (35.5–37.4)	36.1 (35.8–37.2)	0.690
Pre-operative hemoglobin, g/L, (median, IQR)	113 (109–122)	116 (108–122)	0.949
Enteroatmospheric fistula, No. (%)	44 (51.76)	63 (54.78)	1.000
Length of small intestine, cm, (mean ± SD)	270.47 ± 66.77	312.08 ± 72.44	<0.001
Blood loss during definitive surgery, ml, (median, IQR)	1,000 (600–1,200)	1,000 (500–1,200)	0.150
Duration of definitive surgery, min, (median, IQR)	180 (160–210)	180 (180–220)	0.339
Grade of abdominal adhesions ≥ IV, No. (%)	51 (60)	72 (62.61)	0.708
Initial post-operative lactate, mmol/L, (mean ± SD)	3.83 ± 1.45	3.72 ± 1.31	0.607
Post-operative lactate 24 h after DS, mmol/L, (mean ± SD)	2.37 ± 1.36	2.31 ± 1.11	0.734
The amount of red blood cells infused during and 48 h after DS^**^,Unit, (median, IQR)	5 (2–7)	4 (2–6)	0.144
The amount of Albumin infused during and 48 h after DS^***^, g, (median, IQR)	100 (50–120)	100 (50–130)	0.702
Comorbidity, No. (%)			
Hypertensio	3 (3.529)	3 (2.61)	0.701
Diabetes mellitus	3 (3.529)	3 (2.61)	0.701
Chronic hepatitis	1 (1.176)	1 (0.87)	1.000

* *Before admission, the patient received surgical treatment of intestinal obstruction following a previous abdominal surgery*.

** *In order to maintain the Hemoglobin >100 g/L within 48 h after definitive surgery*.

*** *In order to maintain the Albumin >30 g/L within 48 h after definitive surgery*.

### Primary Outcomes

**Figure 2:**
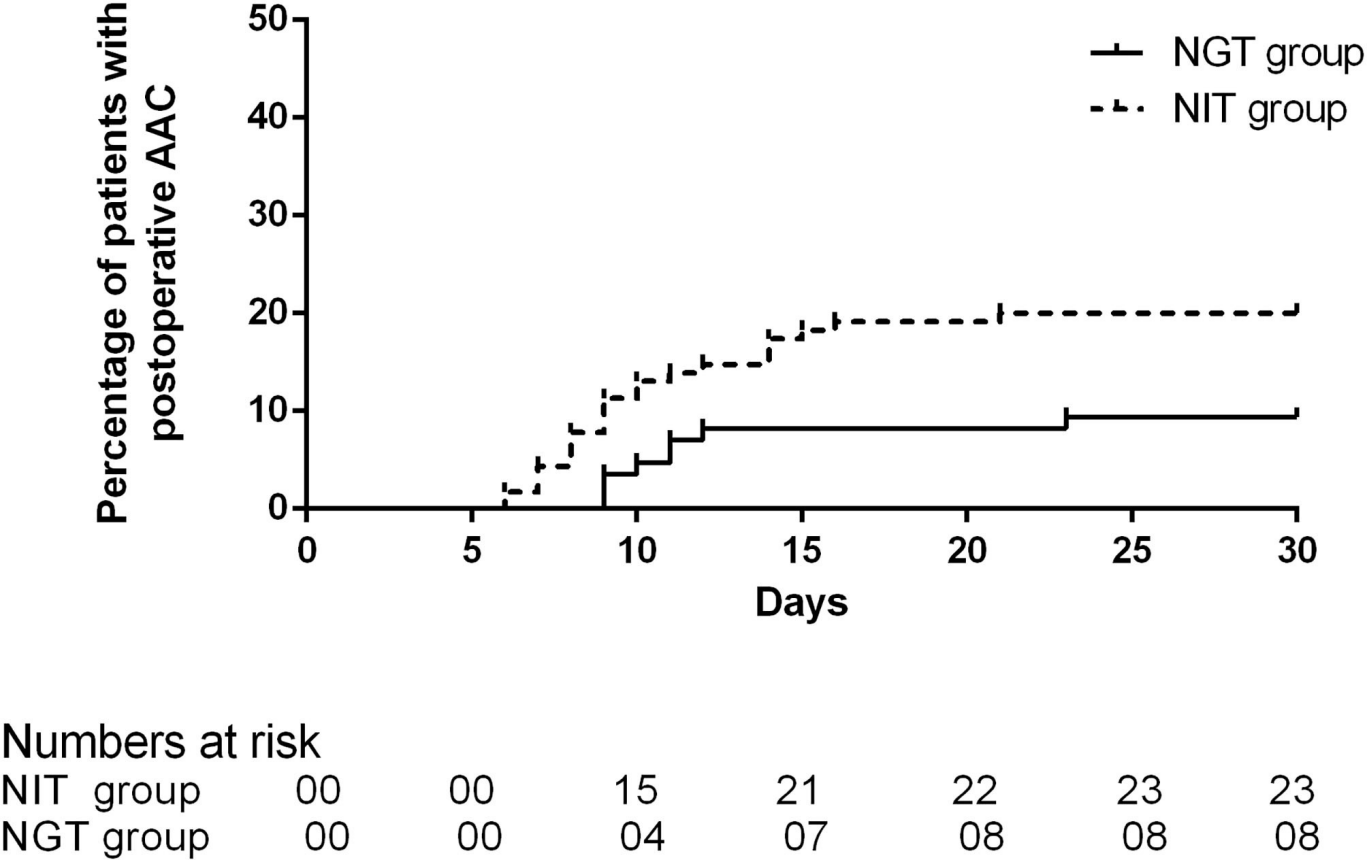
Percentage of patients with post-operative AAC between the two groups.

A total of 31 patients experienced AAC after the DS, and the incidence was 15.5%. Of the 31 patients who developed AAC, 8 (9.41%) were in the NGT group, and 23 were (20%) in the NIT group. The average time of onset was 10 days (IQR 9–11.75) in the NGT group and 9 days (IQR 8–14) in the NIT group. The unadjusted Cox regression showed that patients in the NGT group had a lower incidence of post-operative AAC (log-rank *P* = 0.038; unadjusted HR = 0.438; 95% CI: 0.196–0.980; *P* = 0.045; Table 2; Figure 2) than the patients in the NIT group did. The adjusted Cox regression showed that the pre-operative NGT was associated with a reduction in the incidence of post-operative AAC (adjusted HR = 0.359; 95% CI: 0.139–0.931; *P* = 0.035, Table 3). In addition, the other two factors associated with the post-operative AAC were the number of red blood cells infused during and within the 48 hr after the DS (adjusted HR = 1.245; 95% CI: 1.025–1.512; *P* = 0.027, Table 3) and female sex (adjusted HR = 0.245; 95% CI: 0.104–0.579; *P* = 0.001). The pre-operative CT scan showed that the mean gallbladder volume was 47.98 ± 14.72 mL in the NGT group, lower than that in the NIT group (67.88 ± 11.09 mL, *P* < 0.001).

**Table 2:** Unadjusted regression for post-operative AAC.

	**HR**	**95%CI**	***p***
NGT	0.438	0.196–0.980	0.045
Female	0.277	0.119–0.643	0.003
Age			
<60	Ref		
More than 60	2.859	1.278–6.393	0.011
BMI	0.905	0.662–1.237	0.532
Etiology			
Trauma	Ref		
Obstruction due to previous surgery^*^	1.046	0.425–2.575	0.921
Mesenteric thrombosis	1.248	0.418–3.724	0.691
Spontaneous perforation due to Crohn's disease	0.995	0.371–2.673	0.993
Length from treitz to location of (the first) fistula	1.002	0.996–1.007	0.528
Number of fistula	0.993	0.870–1.234	0.629
High output	1.241	0.571–2.695	0.586
Pre-operative parenteral nutrition required	0.928	0.222–3.891	0.919
Interval from fistula occurred to admission	0.968	0.915–1.024	0.255
Interval from fistula occurred to definitive surgery	0.999	0.978–1.020	0.903
Pre-operative albumin	0.922	0.730–1.166	0.500
Pre-operative hemoglobin	0.999	0.966–1.032	0.540
Enteroatmospheric fistula	1.050	0.517–2.130	0.893
Length of small intestine	0.998	0.993–1.003	0.429
Blood loss during definitive surgery	1.002	1.001–1.003	<0.001
Duration of definitive surgery	1.002	0.995–1.009	0.533
Grade of abdominal adhesions ≥ IV	1.593	0.734–3.461	0.239
Initial post-operative lactate	1.202	0.956–1.514	0.115
Post-operative lactate 24 h after DS	1.034	0.783–1.364	0.814
The amount of Albumin infused during and 48 h after DS^**^	1.016	1.009–1.023	<0.001
The amount of red blood cells infused during and 48 h after DS^***^	1.343	1.199–1.504	<0.001
Comorbidity			
Hypertensio	1.094	0.149–8.026	0.929
Diabetes mellitus	2.622	0.625–10.955	0.188
Chronic hepatitis	3.636	0.495–26.711	0.205

* *Before admission, the patient received surgical treatment of intestinal obstruction following a previous abdominal surgery*.

** *In order to maintain the Albumin >30 g/L within 48 h after definitive surgery*.

*** *In order to maintain the Hemoglobin >100 g/L within 48 h after definitive surgery*.

**Table 3:** Adjusted regression for post-operative AAC.

	**HR**	**95%CI**	***p***
NGT	0.359	0.139–0.931	0.035
Female	0.245	0.104–0.579	0.001
Age			
<60	Ref		
More than 60	1.967	0.841–4.601	0.119
Blood loss during definitive surgery	1.001	0.999–1.003	0.511
The amount of red blood cells infused during and 48 h after DS^**^	1.245	1.025–1.512	0.027
The amount of red blood cells infused during and 48 h after DS^***^	1.005	0.994–1.016	0.413

** *In order to maintain the Hemoglobin >100 g/L within 48 h after definitive surgery*.

*** *In order to maintain the Albumin >30 g/L within 48 h after definitive surgery*.

### Secondary Outcomes

The initial signs and symptoms of post-operative AAC included fever (24/31), abdominal pain (14/31), and vomiting (10/31). Four patients who had AAC (12.90%) had no initial signs and symptoms, but they developed a sudden increase in the white blood cell count. PC was performed in each patient with post-operative AAC. The time interval between the onset of AAC and PC was 20 hr (IQR 18–36). The results of the bile culture are shown in Table 4. *Escherichia coli* was the most common bacteria found in the bile cultures (*n* = 19, 61.29%).

**Table 4:** Characteristics of patients with AAC.

Usage of NGT, *N* (%)	8 (25.81)
Female, *N* (%)	7 (22.58)
Organ injury, *N* (%)	9 (29.03)
Kidney injury	1 (11.11)
Liver injury	7 (77.78)
Kidney injury + liver injury	1 (11.11)
**Age**, ***N*****(%)**	
<60	23 (74.19)
More than 60	8 (25.81)
**Bacterial culture of bile**, ***N*****(%)**	
***Escherichia coli***	
Multidrug or extensive drug resistance	17 (54.83)
Non-multidrug resistant	2 (6.45)
***Klebsiella***	
Multidrug or extensive drug resistance	8 (25.81)
Non-multidrug resistant	1 (3.22)
***Enterococcus faecalis***	
Multidrug or extensive drug resistance	3 (9.68)
Non-multidrug resistant	0
**Signs and symptoms**, ***N*****(%)**	
Fever	24 (77.42)
Abdominal pain	14 (5.16)
Morpheus sign	12 (38.71)
Vomit	10 (32.22)
Simple leukocytosis	4 (12.90)
White blood cell*10^**−9**^**/L**, ***N*****(%)**	
10–20	13 (41.94)
>20	18 (58.07)

**Figure 3:**
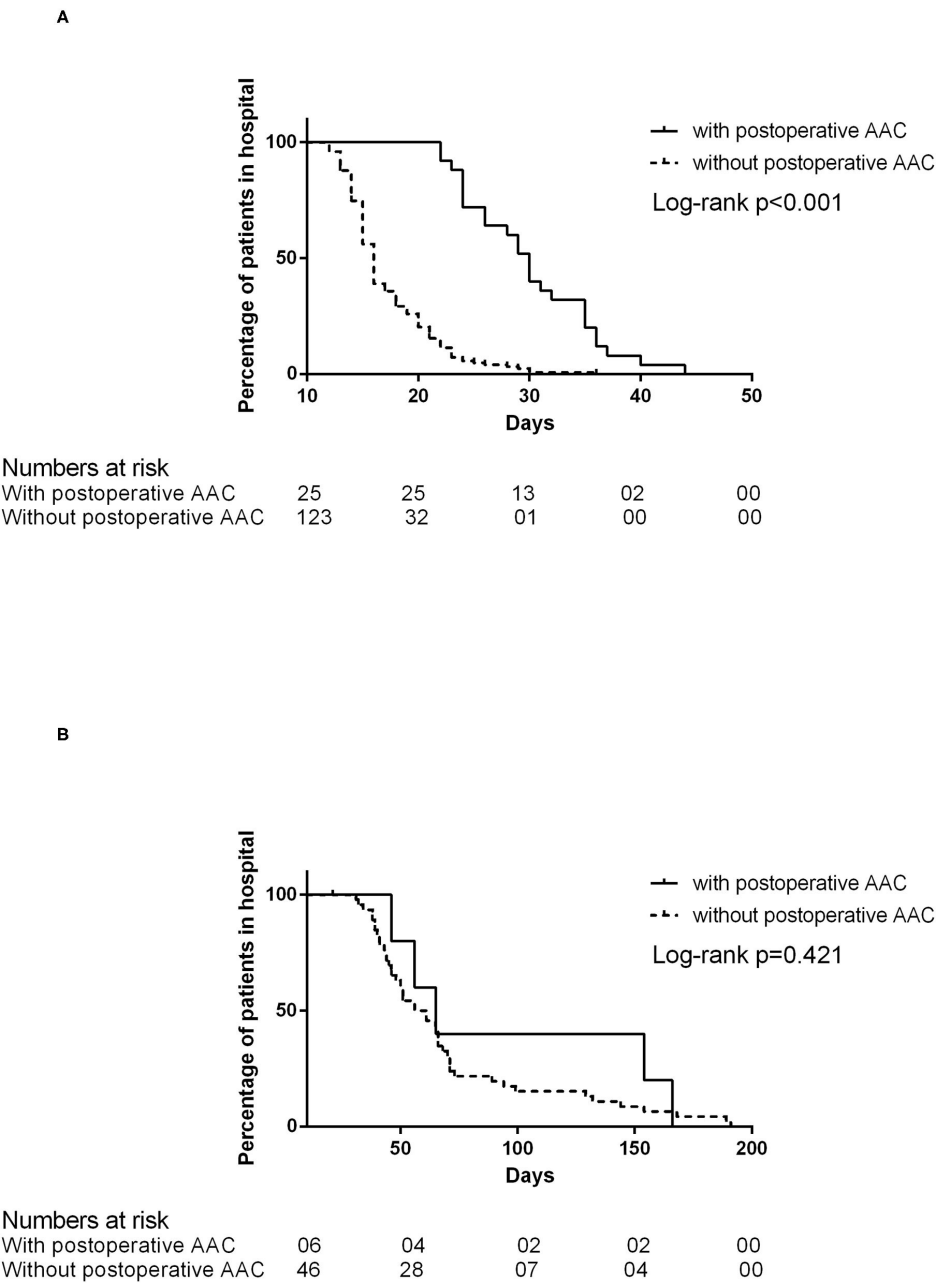
**(A)** The post-operative length of stay in patients without recurrent fistula. **(B)** The post-operative length of stay in patients with recurrent fistula.

Patients would be discharged after they resumed the oral diet. In the patient without recurrent fistula (*n* = 148), the post-operative LOS of the patient was longer if AAC occurred [30 days (IQR 24–35) with AAC vs. 16 days (IQR 14–20) without AAC, log-rank *P* < 0.001; Figure 3A]. However, if the patient had a recurrent fistula (*n* = 52), the post-operative LOS was comparable [60.5 days (IQR 39.75–157) with AAC vs. 58.5 days (IQR 43.7–71.5) without AAC, log-rank *P* = 0.421; Figure 3B)].

### Organ Injury and Mortality

Before puncture, 9 (27.27%) in 31 patients developed organ injury (Table 4). The use of NGT was not associated with organ injury (unadjusted OR = 1.125; 95% CI: 0.212–5.969; *P* = 0.890). However, we found that the detection of *Klebsiella* in the bile culture (OR = 5.625; 95% CI: 1.024–30.904; *P* = 0.047) was a factor associated with organ injury after AAC.

Among the 31 patients that developed AAC, only one (3.23%) died from multiple organ dysfunction syndromes. The only initial symptom of this 56-year-old male patient was fever. It was misdiagnosed as a central venous catheter infection. On the next day of diagnosis, the patient's symptoms did not improve after catheter removal. On the third day of fever, the patient was diagnosed with acute kidney injury, confirmed by a CT scan. The patient received a PC 52 hr after AAC was diagnosed. Multiple drug-resistant *Klebsiella* was isolated from the patient's bile culture.

### Aspiration Pneumonia and Upper Gastrointestinal Bleeding

Five patients developed aspiration pneumonia. Three of the five patients were from the NGT group, and two were from the NIT group (3.53 vs. 1.74%, *P* = 0.652). Only one of the five patients (in the NGT group) had post-operative aspiration pneumonia. This case of aspiration pneumonia occurred on the 17th day after the DS.

Upper gastrointestinal bleeding occurred in five patients: one in the NGT group and four in the NIT group (1.17 vs. 3.48%, *P* = 0.397). Three patients suffered from upper gastrointestinal bleeding before the DS (one in the NGT group and two in the NIT group). Two patients in the NIT group had post-operative upper gastrointestinal bleeding. The two cases of bleeding occurred before returning to EN (on the 4th and 12th days after DS).

## Discussion

This study found that the incidence of AAC in patients with a small intestinal fistula, treated by a DS, receiving pre-operative EN *via* the NGT was less than the incidence of post-operative AAC in those receiving EN *via* the NIT. Only a few previous studies focused on this phenomenon. Thus, the exact pathogenesis of AAC has not been thoroughly understood. Bile stasis and gallbladder wall ischemia might be the main pathophysiological processes of AAC (10). Although gastrointestinal surgery is the most frequently implicated in the etiology of the disease, AAC is quite rare, with an estimated incidence of 0.09% (15). However, the incidence of AAC is relatively high in critically ill patients, ranging from 0.5 to 18% (2). The overall mortality of AAC is 50% (3–5). However, in other studies, the mortality rate varied from 0 to 90% (6, 16–18). Besides, conservative treatments of AAC often yielded poor results. Ganpathi et al. (6) argued that the patient prognosis would be good only if diagnosis and surgical treatment are conducted promptly.

The incidence of AAC in patients who received a DS for small intestinal fistulas found in this study was 15.5%. The incidence might be associated with the clinical characteristics of patients and the disease itself. Patients with a small intestinal fistula are always required to fast and fasting may lead to cholestasis. In addition, the surgical process is complicated and may result in massive blood loss, serious surgical strikes, and prolonged recovery of post-operative perfusion (19). The combined effect of these factors may lead to a high incidence of AAC in patients with a small intestinal fistula. However, the risk factors (volume of blood loss, duration of the surgery, and amount of albumin and blood transfusion needed after the DS) seemed comparable between the NGT group and the NIT group in this study. It was inferred that the difference in the incidence of AAC between the two groups might be related to the routes of EN. In patients with an NGT, the EN was pumped into the stomach before entering the duodenum and jejunum. This route is close to the natural physiology of food digestion. However, for patients with an NIT, the EN was pumped into the jejunum directly. As a result, this method causes relatively little stimulation to the upper gastrointestinal tract. Little stimulation leads to less output. However, this process might lead to the abnormal secretion of gastrointestinal hormones. Cholecystokinin (CCK) is produced by endocrine cells of the intestinal mucosa, which are concentrated at the duodenum and proximal jejunum (11). Besides the actions on the central nervous system, the main functions of CCK are the promotion of gallbladder contraction and bile emptying (11). Patients who have Billroth II or Roux-en-Y reconstructions have a higher incidence of post-operative cholecystolithiasis after surgery than patients who receive Billroth I reconstructions (20–22). This finding suggests that one of the mechanisms leading to post-operative cholecystolithiasis might be the lack of food passage through the duodenum and the change in the pattern of CCK secretion, which reduces the gallbladder contractility and increases gallstone formation (22). The mechanisms of post-operative cholecystolithiasis might be similar to that for AAC in this study: the abnormal secretion of CCK and the abnormal biliary secretion. In addition, the size of the gallbladder before the DS was larger in patients in the NIT group in this study. This might be another evidence indicating that the EN *via* an NIT might exert more negative impacts on bile excretion than the EN *via* an NGT did.

The mortality of AAC is associated with the timing of diagnosis and treatment. The patients with post-operative AAC in our center received prompt PC had a good prognosis. The mortality in our study was only 3.23%. Our experience confirmed that timely management of AAC might be the key in treating severe surgical infections (23).

This study had some limitations. First, retrospective studies are associated with an elevated risk of bias. Thus, the findings may require confirmation by randomized controlled studies in the future. Second, the sample size was fairly small, leading to an elevated risk of bias. The conclusions were based on the findings from this small cohort of 200 patients. However, intestinal fistula is a relatively rare disease with a low incidence rate, and our study was the first to explore the influence of the feeding route on post-operative AAC. Third, our study revealed that female sex was a protective factor for AAC. The result was similar to previous findings that the male sex accounted for 60–80% of patients with AAC (1, 6, 10). However, due to limited understanding of the pathogenesis of AAC, the reason behind the male preponderance of AAC remains unknown and needs further exploration. Fourth, the post-operative fluid management was based on the mean arterial pressure, urine volume, and central venous pressure. Overall, the rehydration strategy was not standardized in our study. Therefore, it was difficult to determine whether dehydration occurred and led to microcirculation insufficiency, followed by AAC. However, the post-operative lactate level was comparable between the two groups, meaning that post-operative fluid management might not significantly influence the microcirculation. In addition, the time interval between the DS and the onset of AAC varied, ranging from 6 to 23 days. It seemed that the onset of AAC was beyond the most likely stage of the hypoperfusion after the DS. As a result, we speculated that fluid management did not have a significant influence on the occurrence of AAC. Some argued that the cholecystectomy should be performed during the DS. However, the condition of each patient was different. Cholecystectomy often represents the separation of more adhesions in the DS for intestinal fistula. Also, the condition of the surrounding tissue during the surgery was unclear, and abrupt cholecystectomy may cause more harm to the patient than benefit. Therefore, we think we need to be cautious when planning a cholecystectomy for the patient.

## Conclusions

Post-operative AAC occurred in 15.5% of patients after the DS for a small intestinal fistula in this study. Pre-operative EN *via* the NGT may reduce the incidence of post-operative AAC.

## Data Availability Statement

The raw data supporting the conclusions of this article will be made available by the authors, without undue reservation.

## Ethics Statement

The studies involving human participants were reviewed and approved by Ethics Committee of Jiangning Hospital and Ethics Committee of Jinling Hospital. The patients/participants provided their written informed consent to participate in this study.

## Author Contributions

YZ and WT provide research objects. XX and RZ collected and analyzed the data. ZY and WT wrote the main manuscript text. XX prepared figures and revised the manuscript. ZY revised the manuscript and designed the research. All authors contributed to the article and approved the submitted version.

## Conflict of Interest

The authors declare that the research was conducted in the absence of any commercial or financial relationships that could be construed as a potential conflict of interest.

## Publisher's Note

All claims expressed in this article are solely those of the authors and do not necessarily represent those of their affiliated organizations, or those of the publisher, the editors and the reviewers. Any product that may be evaluated in this article, or claim that may be made by its manufacturer, is not guaranteed or endorsed by the publisher.
